# The Rise in Single‐Mother Families and Children’s Cognitive Development: Evidence From Three British Birth Cohorts

**DOI:** 10.1111/cdev.13342

**Published:** 2019-11-20

**Authors:** Susan Harkness, Paul Gregg, Mariña Fernández‐Salgado

**Affiliations:** ^1^ University of Bristol; ^2^ University of Bath; ^3^ Universidad de Alcalá

## Abstract

This article assessed changes in the association between single motherhood and children’s verbal cognitive ability at age‐11 using data from three cohorts of British children, born in 1958 (*n* = 10,675), 1970 (*n* = 8,933) and 2000 (*n* = 9,989), and mediation analysis. Consistent with previous studies, direct effects were small and insignificant. For those born in 1958 and 1970 indirect effects, operating through reduced economic and parental resources, were associated with −.107‐*SD* to −.156‐*SD* lower attainment. Differences between the two cohorts, and by children’s age when parents separated, were insignificant. For the 2000 cohort, effect sizes for children born to single mothers did not change significantly (−.112‐*SD*) but attenuated for children whose parents separated in early childhood (−.076‐*SD*) or while of school age (−.054‐*SD*).

Children in single‐mother families typically have poorer outcomes, across a range of measures, than those living with both parents. However, many studies have concluded that, once factors such as low income and poor maternal mental health are accounted for, the impact of family structure on cognitive outcomes is small (see Chapple, 2009, for a review). Yet it is well known that children who grow up in single‐mother families have different socioeconomic circumstances to those living with both parents and this is, at least in part, because they live with a single mother: single motherhood is linked to reduced income, a high risk of poverty, worse maternal mental health, poor parenting practices, and a range of other disruptions, such as home and school moves and multiple family transitions (Hill, Yeung, & Duncan, 2001; McLanahan, 2009). As a result, regression‐based models comparing children in single and two‐parent families which condition on contemporary socioeconomic and psychological characteristics are likely to underestimate the impact of single motherhood on their outcomes. Although prior studies have examined the role that a range of mechanisms play in mediating the relationship between family structure and child outcomes (e.g., Carlson & Corcoran, 2001), they have not previously quantified the magnitude of these relationships. As a result, the indirect effect that single motherhood has on child outcomes remains poorly understood. In this article, using data from three British birth cohorts and structural equation models, we address this gap in the literature. We estimate both the direct and indirect effect of single motherhood on children’s cognitive attainment and assess the relative importance of different mechanisms, including differences, and changes, in economic and parental resources, in driving attainment gaps.

One of the main objectives of this study is to assess how the relationship between single motherhood and children’s cognitive attainment has changed over the last 40‐years. We do so using data from three nationally representative cohorts of British children born in 1958, 1970, and 2000. The direct and indirect effect of single motherhood on children’s cognitive outcomes may have changed for the following reason. First, as the prevalence of single‐mother families has grown, the direct effect may have declined. As Ely, Richards, Wadsworth, and Elliot (1999) investigate in the context of divorce, this may operate through

a reduction in the stigma associated with divorce, an increasing awareness of parents of the potential problems for children, a growing emphasis on both parents remaining in contact with children after separation and the availability of services to assist parents with the postdivorce arrangements for children. (p438)

Similar arguments may apply to single mothers who were never married, never partnered, or widowed. Second, changes in the environment in which children in single‐mother families are growing up in may have reduced the indirect effect of single motherhood on children’s attainment. For example, the indirect effect of single motherhood on child outcomes may have narrowed as women’s employment and earnings have grown, and as state support for single parent families since the late 1990s has increased. At the same time, increased acceptance of alternative family forms may have affected parental inputs; for example, reduced stigma and greater economic independence may have reduced the negative association between single motherhood and mental health (Harkness, 2016), or raised educational aspirations.

A number of prior studies have investigated changes in the relationship between divorce—rather than single motherhood—and children’s educational attainment over time but found little evidence of change (for Great Britain [GB], see Ely et al., 1999; Sigle‐Rushton, Hobcraft, & Kiernan, 2005; for the US see Biblarz & Raftery, 1999). However, these studies use data that are now old (the GB studies use data for children born in 1946, 1958, or 1970; Bilbarz and Rafferty look at outcomes observed in the early 1990s) and, while divorce had become more common over this period, among recent cohorts of British children family structure has continued to dramatically change. Between 1971 and 1998 the share of children in single‐parent families tripled from 7% to 22% before stabilizing (ONS, 2013). At the same time the composition of single‐parent families shifted, with fewer being widows and a growing share of children being born to single mothers. For our samples of children, at age 11, 2% had mothers who were widows (or a quarter of single‐mother families) in the 1958 cohort compared to 1% (or 4% of single‐mother families) in the 2000 cohort. In contrast, 1.6% of children in the 1958 cohort, and 14.1% in 2000, were born to a single mother. These compositional changes were influenced by other shifts; for example, in the 1958 cohort, children born to a single mother were much more likely to be adopted out and “shotgun” marriages were common. Changes over time also reflect shifting attitudes to single motherhood which may influence its observed relationship with child outcomes.

We study how living with a single mother affects children’s cognitive ability, an outcome which is strongly related to a range of later life outcomes, including school leaving qualifications, earnings, occupational attainment, crime, substance abuse, and mental health (Fergusson, Horwood, & Ridder, 2005; Heckman, Stixrud, & Urzua, 2006). Our outcome measure is verbal ability, a measure which is available for each of the cohorts and therefore allows us to examine changes over time. It notable, however, that prior studies have shown a stronger relationship between family structure and children’s emotional outcomes (Cheng, Dunn, O'connor, & Golding, 2006) which is influenced by different pathways (Carlson & Corcoran, 2001). Outcomes are measured at age 10 (1970 cohort) or 11 (1958 and 2000 cohort), the latest age for which comparable data are available and an important milestone in children’s education as they transition from primary to secondary school. Children are assumed to have experienced living with a single mother if their biological father does not live in the same household. Our focus is on single mothers, rather than single parents, because single fathers have very different socioeconomic characteristics and parenting styles (Bronte‐Tinkew, Scott, & Lilja, 2010). Moreover, in the cohorts studied, fewer than 1.5% of all children, or 4% of those experiencing single parenthood, had spent any time with a single father by age 10/11. We allow the effect of single motherhood on children’s attainment to vary by the age at which children first experience living in a single‐mother family because the child’s developmental stage at the time of parental separation may directly, and indirectly, influence their outcomes.

Using structural equation models, we provide important new insights into the drivers of childhood disadvantage among children in single‐mother families and how they have changed over time. The results have potentially important policy implications: for example, if deficits in cognitive attainment are largely a result of reduced economic resources then this is where policy should focus, but if deficits result mainly from changes in parenting behavior policies to boost income are unlikely to be effective.

## Literature Review

**Figure 1:**
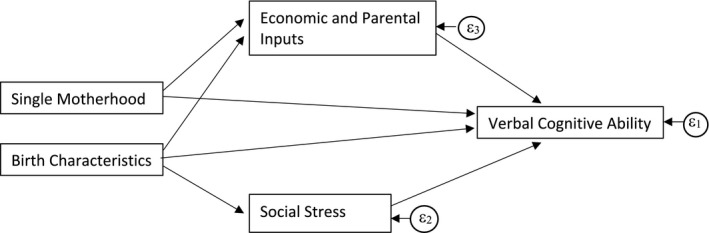
Mediation model of the effect of single motherhood on cognitive outcomes.

We develop a model of child cognitive development which assumes that single motherhood influences children’s cognitive outcomes directly and indirectly. The “direct” effect is assumed to result from family structure per se, with father absence linked to poor discipline, a lack of social control, and social stigma. In assessing the “indirect” effect of single motherhood on child outcomes, we focus on theories from economics and sociology and assume that single motherhood influences children’s outcomes via two mediating pathways. The first pathway comes from changes, and differences, in economic and parental resources, compared to living with both parents. This pathway is chosen because, as Biblarz and Raftery (1999) note, “almost all existing theory around the consequences of family structure for children centers around the relationship between family type and resources” (p323). The second pathway comes from the literature on social stress which suggests that family instability has a damaging impact on children (Amato, 2000). Family change—including fathers’ departure and mothers’ repartnering—are associated with parent and child stress and further disruptions, such as home and school moves, affecting children’s development (Cherlin et al., 1991). In addition, single‐mother families may have different observable characteristics (such as mothers age, education, or child birth weight) at the time of birth than families who do not separate. These differences, rather than single motherhood per se, may affect children’s verbal cognitive outcomes. We adjust for these confounding variables. The pathways between single motherhood and children’s cognitive outcomes are illustrated in Figure 1. Next, we summarize the relevant literature on these pathways, consider how children’s age at the time of parental separation might affect these relationships and assess why these associations may have changed over time. Finally, we discuss how the nonrandom selection of single‐mother families may affect our estimates.

### The Direct Effect of Single Motherhood and Father’s Absence on Children’s Cognitive Outcomes

Theory presumes that family structure has a direct causal effect on children’s cognitive outcomes. One reason for this is that father absence reduces the amount of time families have to invest in children and this is assumed to be detrimental to their outcomes (McLanahan, 2004). However, it is not just the amount of time available for children that matters. Social control theory suggests that the absence of a father figure in the household has adverse consequences for children because the number of adults available to supervise children falls and, even when there are other adults in the household (such as step or grandparents), they are likely to have a more distant relationship to the child and therefore exert less control (Hill et al., 2001). Other authors emphasize the quality of father–child relationships, rather than the amount of time children spent with their fathers. Cabrera, Tamis‐LeMonda, Bradley, Hofferth, and Lamb (2000) argue that fathers play a unique role in their children’s development which cannot easily be substituted for by maternal care. For example, their masculine qualities encourage children to be competitive and take risks. Empirical studies further indicate that father involvement benefits children’s cognitive outcomes (Huerta et al., 2013) and plays an important role in compensating for low‐quality maternal parenting (Martin, Ryan, & Brooks‐Gunn, 2010). Conversely, father absence is linked to truancy, delinquency, crime, and poor school performance, particularly for boys from low‐income families (Autor, Figlio, Karbownik, Roth, & Wasserman, 2016). Of course, fathers may be involved with their children even if they do not live in the same household. Evidence on how nonresident fathers’ involvement affects child outcomes, however, suggests it may have a limited effect. Amato and Gilbreth’s (1999) meta‐analysis of 63 studies finds no evidence that frequency of children's contact with their father is associated with improved outcomes, although there is a positive association between fathers’ payment of child support and attainment.

Over recent decades father’s involvement in child care has increased among two‐parent families (Cabrera et al., 2000). In the United Kingdom and elsewhere this has been encouraged by a range of policy initiatives such as the introduction of paternity leave and shared parental leave (Huerta et al., 2013). At the same time, more children are growing up in single‐mother families with little or no father contact. The growing gap in paternal involvement as a result of family change is strongly graded by socioeconomic status (SES): fathers with few economic resources and who are less able to financially support their families are least likely to be involved in children’s care (McLanahan, 2004). These growing differences are expected to lead to increased attainment deficits for children in single‐mother families over time.

The age at which parental separation takes place may influence children’s attainment. If father involvement leads to better outcomes for children, single motherhood may be more damaging for those born to a single mother or experiencing single motherhood while young as fathers’ inputs over the course of their childhood are likely to be minimal. This is particularly the case because children whose parents separate while they are very young are less likely to have father contact (Hetherington & Kelly, 2002). For children whose parents separate while they are of school age, time spent at school rather than home may lead to fewer detrimental effects.

While it is difficult to separately identify the effect of growing up in a single‐mother household from that of father absence there are other reasons to believe that living with a single mother may affect children’s outcomes. If children in single‐mother households face stigma this may affect their cognitive outcomes, for example, because teachers have lower expectations of them (Feinstein & Symons, 1999). Stigma may, however, have reduced over time as single‐motherhood has become more common, reducing attainment gaps.

While, on average, fathers’ presence at home may be beneficial for children, this is not always the case. Separation may be less detrimental to children than continuing to live with both parents if fathers exhibit antisocial traits or there is a high degree of parental conflict (Amato, 2005; Musick & Meier, 2010). Where parental conflict is high, children born to single mothers or experiencing parental separation while young may be exposed to less parental conflict, and early parental separation may be more beneficial to their outcomes than those which occur in later childhood.

### Skill Formation: Economic and Parental Inputs

Living in a single‐mother family is associated with a host of other childhood disadvantages. Of particular significance is its association with reduced income and higher risk of poverty (Page & Stevens, 2004) both of which are linked to lower cognitive attainment (Gennetian, Castells, & Morris, 2010; Holmes & Kiernan, 2013). Income is assumed to influence child outcomes through two channels. First, economic theories suggest that higher income allows parents to invest more in children’s development. Children in higher income families may have better access to material goods and a superior home learning environment (e.g., because they have greater access to books, toys, and other educational resources). They are also more likely to participate in educational activities, such as trips and outings, and their parents are able to access higher quality child care and education (Duncan, 2005). Second, psychological theories emphasize the effect of income on parental stress, which in turn influences parenting styles, parent–child relationships, and child well‐being (McLoyd, 1998). The importance of parental inputs are increasingly emphasized by economists too; for example, Heckman argues “conventional measures of family disadvantage [..] such as ‘broken home’ or family income, are very crude proxies for the real determinants of child outcomes” (Heckman, 2008, p305) with the quality of the nurturing environment and positive parenting particularly important for moderating the influence of poverty and family disadvantage on cognitive attainment (Kiernan & Mensah, 2011).

Theory and evidence on the effect of income on child outcomes underline some further important associations. First, the impact of income on child development is highly skewed, with increases in income having larger effects on children from low‐income backgrounds. Second, child outcomes bear a stronger correlation with permanent rather than transitory measures of income (Duncan, Ziol‐Guest, & Kalil, 2010) and poverty (Dickerson & Popli, 2016). This may reflect income smoothing over the life‐cycle, with families being able to adapt to temporary income shocks than persistent low income. Third, there is growing evidence that poverty and low income have larger effects when children are young (Reardon, 2011). This is consistent with economic theories of skill development, which suggest that investments in the early years are critical because of their influence on returns to investments in later years (Heckman, 2006); and with theories that emphasize the impact of early childhood environments on biological development (Shonkoff, 2010). This suggests three reasons to expect the effect of single motherhood on child outcomes, mediated through low income, to be greater for children born to a single mother or who experience early parental separation. First, children born to a single mother or experiencing parental separation during early childhood may face prolonged exposure to low income during their childhood, with greater adverse effects. Second, the predicted effect of low income on child development is larger in early childhood and, third, Heckman’s model of skill acquisition suggests that, as early inputs have a strong effect on the productivity of later investments, adverse circumstance during early childhood will have a cumulative effect on later outcomes.

Family breakdown may also affect parental inputs. Studies report that single motherhood is associated with poorer home environments and parenting behaviors including a lack of routine, harsh discipline and lower levels of parental supervision (Amato, 2005; McLanahan, 2004). Single mothers are also at high risk of poor mental health (Targosz et al., 2003), with maternal depression associated with lower levels of child cognitive and emotional well‐being (Kiernan & Huerta, 2008).

The indirect effect of single motherhood on child outcomes, and in particular that mediated by reduced income, may have changed over time as female employment has grown. Between 1971 and 1980 (close to when mothers’ employment in the 1958 and 1970 cohorts are observed) female employment grew three percentage points, to 56% (ONS, 2019). At this time, single and partnered mothers were equally likely to be employed, with single mothers more likely to work full‐time than those with partners because rules on claiming out‐of‐work benefits strongly disincentivized part‐time work. The late 1980s and 1990s (a period which our cohorts do not cover), however, saw the employment rate of single mothers fall far behind that of mothers in couples; by the early 1990s, 40% of single mothers were working compared to over 60% of mothers with partners (Gregg & Harkness, 2003). As a result, children in single‐parent families faced a high risk of poverty. Under New Labour a package of “welfare reform” policies were introduced with the aims of making work pay and reducing rates of child poverty. For single parents, a 70% employment target was set with policies to achieve this including the New Deal for Lone Parents (which supported lone parents find work), the introduction of tax credits and increased means‐tested support for child care. These reforms were associated with large increases in single‐mothers’ employment (Gregg, Harkness, & Smith, 2009), much of which was part‐time. New Labour also emphasized early years education, and policies including the National Child Care Strategy and the roll‐out of Sure Start children’s centers which may have disproportionately affected the cognitive attainment of low‐income children, including those in single‐mother families. Finally, the influence of income on attainment may have changed; research for the United States shows cognitive attainment gaps between poor and nonpoor children have grown since the 1970s (Ziol‐Guest, Duncan, & Kalil, 2015) and this may also affect the attainment of children in single‐mother families.

### Family Change and Social Stress

Divorce and separation are stressful life course events which negatively affect children’s emotional well‐being (Amato, 2000). Stress theory emphasizes the role that family change, rather than family structure per se, plays in driving child outcomes. While children may adapt to change over time, further or repeated exposure to family change will have increasingly damaging consequences. In particular, stress theory predicts that divorce and separation, as well as repartnering, are damaging for children because family members’ roles within the household, and parent–child relationships, change (Berger & Bzostek, 2014). As a result, living with a single mother, even for a prolonged period, may be less detrimental for children than further family change. In recent years there has been growing concern in the United States about children’s exposure to multiple family transitions, and the growing prevalence of multipartner fertility, with empirical research suggesting it has a cumulative negative effect on children’s attainment (Fomby & Osborne, 2017). This may vary with the age at which parents separate as children born to single mothers, or whose parents separate when they are young, are more likely to be exposed to further family change and multiple family transitions (Osborne & McLanahan, 2007). Parental separation is linked to a range of other disruptions, also associated with stress, such as school and home moves (Hill et al., 2001). These changes may have a greater impact on older children although, as children adapt to change over time, effects may be largest around the time of separation.

While repartnering is associated with stress, it is also linked to an increase in the availability of financial and parental resources (Page & Stevens, 2004) which benefit children. Evidence suggests, however, that spending on children is lower in stepparent families and the impact of increased income on children’s outcomes may therefore be muted (Case, Lin, & McLanahan, 2000). Moreover, repartnering is not associated with improved cognitive outcomes, with outcomes for children in single and stepparent families being broadly similar (Musick & Meier, 2010).

### Selection into Single Motherhood and Child Outcomes

A major challenge for identifying the effect of single motherhood on child outcomes is selection bias as the characteristics influencing the likelihood of becoming a single mother correlate strongly with child outcomes. For example, the personality traits that drive the risk of becoming a single parent also influence parenting and child attainment (Amato, 2005). Many of these characteristics are unmeasured so identifying causal associations is difficult. To account for sample‐selection bias, studies of children of single parent families have used sibling differences, fixed effect, or instrumental variables (see Chapple, 2009). However, as Moffitt (2005) notes, the identification assumptions used in these studies tend to emphasize the internal validity of results while frequently compromising generalizability. For example, sibling studies exclude many single‐parent families who have just one child while studies using fixed‐effects require cognitive outcomes to have been measured prior to parental separation and are of little relevance to those whose parents separate during early childhood, before cognitive outcomes are first measured. Traditional tools to correct for selection bias cannot therefore easily deal with selection issues for a large share of children experiencing single parenthood (McLanahan, Tach, & Schneider, 2013). In our data, children experiencing early parental separation account for more than half of those whose parents separated by age 11 in all cohorts. Yet, as we show later, these children have particularly low attainment and are those for whom we might otherwise be most concerned. Of course, differences in children’s cognitive attainment by age of parental separation may reflect differential selection into single motherhood by child age, with those experiencing single motherhood during early childhood particularly adversely selected. In this case, the negative association between child outcomes and age at the time of parental separation may reflect these unobserved characteristics. Selection into single motherhood may also have changed over historic time although, a priori, it is hard to predict in what direction. For example, as single motherhood has become more common it may be less concentrated on the most disadvantaged. Alternatively, it may have become increasingly concentrated on those women with the fewest opportunities and, if this is the case, attainment gaps may have widened.

### Current Study: Mediation Analysis

This study aims to identify the “direct,” “indirect” and “total” effect of single motherhood on children’s cognitive outcomes and show how they have changed over 40 years. We do so by quantifying the influence of single motherhood on economic and parental inputs and assessing their role in mediating reduced cognitive scores. Outcomes are measured using standardized test scores. Differences over time are interpreted as changes in the performance of children in single‐mother families relative to the mean. While previous studies have shown that the association between single motherhood and child outcomes is considerably reduced once factors linked to single motherhood, such as lower income or worse maternal mental health, are accounted for, to our knowledge none have quantified the size of these indirect  effects. Examining the different pathways by which changes in children’s outcomes occur also allows us to understand the mechanisms influencing test scores and show how they have changed.

## Methods and Data

### Data and Sample

We draw on data from three British birth cohort studies that follow children born in 1958, 1970 and 2000 over time. The studies are large representative surveys of children growing up in GB designed to allow comparisons across cohorts (Centre for Longitudinal Studies, 2019). The National Child Development Study (NCDS) collected data on children born in a single week in 1958 and surveyed them again at age 7, 11, 16, and into adulthood. The British Cohort Study collected data for children born in a week in 1970 and followed them up at age 5, 10, 16, and in adulthood. The 2000 survey, the Millennium Cohort Study (MCS) followed a sample of babies born between September 2000 and January 2002 with interviews at 9 months, 3, 5, 7, 11, and 14. It collected information from the UK households with booster samples of children from disadvantaged families, ethnic minorities, and those living in Scotland, Wales, or Northern Ireland (NI). For comparability across cohorts we exclude the NI sample. All surveys collected detailed information on children’s cognitive development, economic, and family characteristics, health, and well‐being. We focus on cognitive outcomes at age 10/11 and limit our study to children living with their biological mother (adopted children, those in foster care, living with a single father, or other family members are excluded).

The 2000 cohort sample for GB has 11,660 observations at age 11. Of this sample, we exclude 297 children who do not live with their biological mother, 248 children for whom we do not have data on test scores, and 866 children for whom we have insufficient data to construct family histories. As is common in the literature on child development, we exclude a further 260 twins or triplets present in the survey at age 11. All other missing variables are handled using maximum likelihood methods. Our resulting sample size is 9,989. For the 1958 and 1970 cohorts, samples are similarly constructed, resulting in sample sizes of 10,675 and 8,933 respectively. For the 2000 cohort, to ensure findings are representative of the population, we apply overall survey weights [eowt2] and use the Stata “svy” commands to take account of attrition bias and the survey’s design, including clustered sampling (Mostafa, 2014; Plewis, 2007).

We do not use weights for the earlier cohorts as studies show they do not improve estimates or standard errors (Hawkes & Plewis, 2006; Mostafa & Wiggins, 2015). For simplicity, we describe outcomes as occurring at age 11 (rather than 10/11) and to events that occur before or after 7 (rather than 5/7).

### Outcome Measures: Cognitive Attainment at Age 10/11

Verbal cognitive ability, which is available in each of the cohorts, is our main outcome measure and has been widely used in other studies using the same data to conduct cross‐cohort comparisons (e.g., Goisis, Özcan, & Myrskylä, 2017; Henderson, Richards, Stansfeld, & Hotopf, 2012). For the 1958 cohort, the verbal ability test forms part of the General Ability Test and is administered by teachers when children are 11. The test has 40 items, with children given a word sequence and asked to select one that continues a pattern. The 1970 cohort verbal ability test is derived from the 21‐item word similarity subscale of the British Ability Scales (BAS; first edition). It is conducted by teachers and measured at age 10. The 2000 verbal ability test scores are derived from the 37‐item verbal similarity subscale of the BAS (second edition) and administered by the interviewer at age 11. In each of the cohorts the tests are considered a good proxy for IQ (see Douglas, 1967 on measures in the NCDS; Elliott, Murray, & Pearson, 1979 on the BAS measure; Elliott, 1996, for the BAS measures in the MCS). To take account of differences in the tests, and to adjust for age differences at the time of taking the test, age‐adjusted measures of attainment are calculated by regressing tests score on age, measured in months, at the time of the test. The regressions’ residuals are then normalized to have mean zero and standard deviation one. The resulting scores are comparable both within and between cohorts.

Reading and math test scores are also available in the 1958 and 1970 cohorts. In the 2000 cohort, data are only available for children in English state schools taking the government administered Key Stage 2 tests. To test our findings robustness, we compare our estimates for verbal test scores for children in GB to those for math and reading in England.

### Single Motherhood

All children living with their mother but not their biological father at age 11, when our outcomes are measured, are assumed to have experienced single motherhood. We distinguish between children according to their developmental stage at the time parents separate. Specifically, we examine children born to single mothers (“birth” single mothers); those experiencing single motherhood during early childhood, defined as those living with both biological parents at birth but whose parents separated by age 7 (“early” single mothers); and those experiencing single motherhood during middle childhood, defined as living with both biological parents at birth and early childhood, and with their biological mother but not their biological father at 11 (“middle” single mothers). Children living with single mothers and those whose mothers have repartnered are both coded as having experienced single motherhood. The latter part of our analysis adds a further binary variable to indicate the presence of a stepparent.

In the 1970 cohort we are not able to observe whether mothers are cohabiting with the child’s father at the time of birth as only four marital statuses are reported (single, widowed, divorced, or separated). We assume those who were single at birth but living with the child’s father at 5 had lived with the child from birth (*n* = 143). For consistency across cohorts, we similarly recode the small number of women who report being single at the time of birth but are later observed living with the child’s biological father as having lived together from birth (*n* = 170 and *n* = 180 for the 1958 and 1970 cohorts respectively). For the 2000 cohort we have an additional wave of data for children aged 3. We examine an additional group for this cohort, those living with both biological parents at 3 but whose parents had separated before 7. As we explain in the following section, analyzing this group allows us to test the robustness of our results for “early” single motherhood.

### Other Covariates

Our models include two sets of covariates; those observed at the time of birth (and therefore, for those that separate, before the transition to single motherhood) and mediating variables observed at age 11 (after single motherhood has occurred). Details of the variables used in each cohort, and their definitions, are in Table S1. Because we are interested in examining changes over historical time, we focus on variables available across cohorts. For all children, we observe mother and child characteristics at birth. The child characteristics we condition on are gender, older siblings, ethnicity, and low‐birth weight. Mother characteristics are age, education, SES, and smoking during pregnancy. We also control for region of residence. As data on fathers are missing for children born to single mothers the reported results do not control for their characteristics. However, adding father controls where available contributes little additional explanatory power because mother and father characteristics are highly correlated.

Our second set of covariates are mediating variables, measured at age 11, which we describe under the three broad headings: economic resources, parental resources, and disruptions leading to social stress. The economic circumstances considered are mothers’ employment, home ownership, and logged equivalized income. In the 1958 survey, as income is not reported at age 11, we controlled for whether the respondent reported financial difficulties. Parenting has changed considerably over the last 40 years and consequently the measures of parenting that are available and their interpretation has changed. Parental aspirations, for example, are important for children’s educational outcomes (Feinstein & Symons, 1999). Yet, while mothers in the 1958 cohort may have aspired for their children to stay at school beyond 15, today most parents expect children to stay on at school and many hope they will go to university. Similarly, the meaning of library visits or playing sport differs for children across cohorts. The first part of our analysis therefore focuses on measures that are broadly comparable across surveys. These inputs are mother’s aspirations for her child (expectation of staying on at school beyond the minimum leaving age for those in the 1958 and 1970 cohorts and of going to university in the 2000 cohort) and mothers’ mental health (a dummy variable for depression). For disruptions leading to social stress we include measures of the number of schools attended by 11. We also examined home moves but as it did not show any correlation with attainment it is excluded from the final models. Both home and school moves may take place for a range of reasons, and the effect on child outcomes may not always be deleterious. As a result, the estimated impact of school moves on cognitive outcomes is likely to underestimate the negative impact of school moves resulting from family instability on child outcomes. We also assess the impact of repartnering on children’s outcomes.

Further parenting measures are available for each cohort, although comparability is limited. For the 1958 cohort we have information on library visits, whether the child goes on outings with their parents and playing sport. The 1970 cohort contains this information as well as details on trips to museums and whether the child plays a musical instrument. In the 2000 cohort information on library visits, playing an instrument, outings with parents, playing sport, whether the child has regular bedtimes, and having rules on the time the child spends on the computer is reported. There is also information on whether the child receives help with homework but, as it is negatively correlated with child outcomes (as children who struggle at school may be more likely to receive help) and shows no relation with single motherhood it is not included in the final models. Finally, for children whose parents separate in middle childhood we have measures of cognitive attainment at 7 or 5. In the 2000 cohort, we also have age‐3 attainment. Including these standardized test scores as additional controls allows us to account for differences in attainment prior to parental separation.

### Analytic Strategy

To estimate the direct and indirect influence of single motherhood on children’s cognitive outcomes we use structural‐equation models (SEM). The unmediated model is expressed as:

(1) $$A_{\mathrm{ic}}=\alpha_{c}+\gamma_{c}{\text{LM}}_{\mathrm{ic}}+\mu_{c}X_{\mathrm{ic}}+\varepsilon_{c},$$

where *A_ic_* represents child *i* in cohort *c*’s cognitive attainment; LM*_ic_* is a vector of indicator variables for whether the child experienced single motherhood at birth, during early or middle childhood; and *X_ic_* is a vector of exogenous control variables, observed prior to single motherhood. γ*_c_* gives the “total effect” of single motherhood on children’s outcomes.

The mediated model is given by:

(2) $$A_{\mathrm{ic}}=\alpha_{0c}+\beta_{\mathrm{mc}}\sum_{m=1}^{n}Z_{\mathrm{mic}}^{{}^{\prime }}+\gamma_{0c}{\text{LM}}_{\mathrm{ic}}+\mu_{0c}X_{\mathrm{ic}}+\varepsilon_{0c}$$

$$Z_{1ic}=\alpha_{1c}+\gamma_{1c}{\text{LM}}_{\mathrm{ic}}+\mu_{1c}X_{\mathrm{ic}}+\varepsilon_{1c}$$

$$Z_{\mathrm{nic}}=\alpha_{\mathrm{nc}}+\gamma_{\mathrm{nc}}{\text{LM}}_{\mathrm{ic}}+\mu_{\mathrm{nc}}X_{\mathrm{ic}}+\varepsilon_{\mathrm{nc}},$$

where *Z_mic_* are the mediating pathways (1 to *n*) through which single motherhood affects child *i* in cohort *c*’s attainment which are also affected by *X_ic_*. The mediating pathways are assumed to be independent of one another and the error terms $\varepsilon_{0c},\varepsilon_{1c},\;\cdots \;\varepsilon_{\mathrm{nc}}$ randomly distributed. The vector of coefficients, γ_0c_ show the direct effect of single motherhood on children’s cognitive attainment. Baron and Kenny’s (1986) multiple mediation methods are used to calculate indirect effects using the product method with mediating variables having an indirect effect on cognitive outcomes calculated as γ*_mc_* × β*_mc_*. The sum of the direct and indirect effects equals the total effect in Equation 1 (γ_0_ *_c_* + (γ*_mc_* × β*_mc_*) = γ*_c_*; see Kenny, 2018).

As our dependent variable is normalized (0,1) and single motherhood is a binary variable, coefficients and effect sizes are expressed as standard deviations of verbal ability and are comparable across models (Lachowicz, Preacher, & Kelley, 2018). To test for changes in total and indirect effect sizes across cohorts we pool data and conduct pairwise estimations for: (a) 1958 versus 1970, (b) 1958 versus 2000 and (c) 1970 versus 2000. The control variables are fully interacted with cohort and the coefficients estimated are identical to those computed when separate models are run. Standard errors are robust and, for the indirect and total effects, bootstrapped with 200 replications. Models are estimated using SEM software in STATA with maximum likelihood methods to handle missing data (Allison, 2003).

A potential concern is that, because single‐mother families are not randomly selected, unobserved heterogeneity may influence our results. For children experiencing single motherhood for the first time in middle childhood, because we observe cognitive attainment prior to parental separation, we are better able to account for endogeneity bias. Including age 7 attainment, denoted by *A_ic_*_7_, our model becomes a partial fixed‐effects model, with previous test scores capturing unobserved differences between families and children (Hanushek, 1992). For the mediating variables, we similarly condition on prior conditions (denoted by *Z_ic_*_7_) as these estimates may also be subject to selection bias (Equation 3).

(3) $$\begin{array}{cc} A_{\mathrm{ic}}=\alpha_{0c}+\omega_{c}A_{ic7}+\beta_{\mathrm{mc}}\sum_{m=1}^{n}Z_{\mathrm{mic}}^{{}^{\prime }}+\gamma_{0c}{\text{LM}}_{\mathrm{ic}}\\ & \;+\mu_{0c}X_{\mathrm{ic}}+\varepsilon_{0c} \end{array}$$

$$Z_{1ic}=\alpha_{1c}+\kappa_{1c}Z_{ic7}+\gamma_{1c}{\text{LM}}_{\mathrm{ic}}+\mu_{1c}X_{\mathrm{ic}}+\varepsilon_{1c}$$

$$Z_{\mathrm{nic}}=\alpha_{\mathrm{nc}}+\kappa_{\mathrm{nc}}Z_{ic7}+\gamma_{\mathrm{nc}}{\text{LM}}_{\mathrm{ic}}+\mu_{\mathrm{nc}}X_{\mathrm{ic}}+\varepsilon_{\mathrm{nc}}.$$

Finally, for the 2000 cohort, we have a measure of attainment and inputs at age 3, allowing us to also see how unobserved heterogeneity influences the results for a subgroup of children experiencing single motherhood during early childhood (those who lived with both natural parents at age 3 but only with their mother at 7).

## Results

### Descriptive Statistics

**Figure 2:**
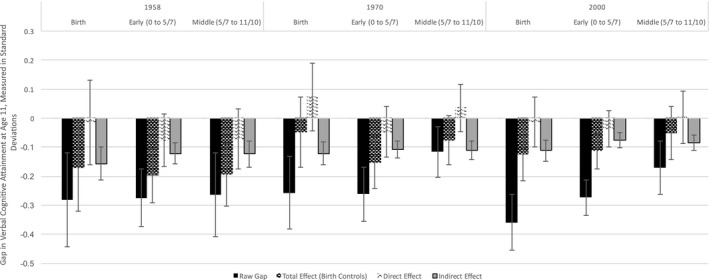
The association between the experience of living with a single mother in early, middle and late childhood and verbal cognitive test scores at age 11 (measured in standard deviations) in 1958, 1970 and 2000: Raw gaps and total, direct and indirect effects. *Note*. For the 1958 and 2000 cohorts, early childhood is defined as parental separation before age 7, in the 1970 cohort, it is separation before 5. Middle is defined as separation between age 7 and 11 in the 1958 and 2000 cohorts and between 5 and 10 in the 1970 cohort.

Figure 2 shows raw gaps in verbal cognitive ability at age 11 between children who had lived with a single mother and those that have not. In each cohort living with a single mother is associated with lower verbal cognitive ability. For those born in 1958 deficits, measured in 1969, were around −.27‐*SD* regardless of the child’s age when parents separated. For the 1970 cohort attainment deficits, measured in 1980, were unchanged for children born to single mothers or whose parents separated in early childhood (around −.26‐*SD*) but smaller for those whose parents separated in middle childhood (−.12‐*SD*). In the 2000 cohort, we see sharp grading in attainment deficits by child age at the time of parental separation with deficits, measured in 2011, for those born to single mothers widening to −.36‐*SD*, remaining at −.27‐*SD* for those whose parents separated between 0 and 7, and narrowing to −.17‐*SD* for those whose parents separated between 7 and 11.

Table 1 reports descriptive statistics for selected mother’s characteristics at the time of the child’s birth (mean values for other controls, except region, are in Table S2). The share of children experiencing single motherhood before the age of 11 grew from less than 10% of those born in 1958 to almost 40% of those born in 2000, with a growing share of children being born to single mothers. Women who became single mothers were typically younger at the time of their child’s birth than those who remained in couples, with larger differences among more recent cohorts. In the 2000 cohort, among children whose parents lived together at age 11, 47% had mothers over 30 at birth and 5% had mothers under 21. For those whose mothers were single at birth, respective figures were 18% and 38%. We observe a similar polarization in mothers’ educational attainment and smoking behavior during pregnancy by family type suggesting single mothers, and particularly birth single mothers, are becoming increasingly negatively selected on observable characteristics. The contribution of these differences in characteristics, observed prior to birth, to the observed deficits in attainment of children in single‐mother families are shown in Figure 2. After accounting for parent and child characteristics at birth attainment deficits are smaller; among those born in 1958, deficits are significant and of a similar magnitude for those born to single mothers and experiencing later parental separations (deficits are between −.17‐*SD* and −.20‐*SD*). In the 2000 cohort attainment deficits show a gradient by the age of children at the time of parental separation: those born to single mothers or whose parents separate in early childhood have significant attainment gaps (of around −.12‐*SD*), while for those children whose parents separate between 7 and 11 there is no significant difference between their attainment and that of children in two‐parent families. These changes are discussed in the following section.

**Table 1:** Descriptive Statistics

	1958 cohort				1970 cohort				2000 cohort			
	Both parents at 11	Single mothers at birth	Single mothers, early childhood	Single mothers, middle childhood	Both parents at 11	Single mothers at birth	Single mothers, early childhood	Single mothers, middle childhood	Both parents at 11	Single mothers at birth	Single mothers, early childhood	Single mothers, middle childhood
	(1)	(2)	(3)	(4)	(5)	(6)	(7)	(8)	(9)	(10)	(11)	(12)
Mother’s characteristics at birth												
Age: mean age	27.7	24.9	27.3	27.4	26.2	22.7	24.3	24.8	30.0	24.4	26.1	28.8
Under 21 at birth	13%	41%	18%	19%	18%	55%	30%	29%	5%	38%	23%	9%
Over 30	28%	21%	29%	28%	20%	12%	9%	13%	47%	18%	23%	35%
Education												
Left school at/before minimum leaving age	74%	82%	79%	80%	66%	74%	73%	65%				
Left school after 19					6%	2%	4%	5%				
Qualifications												
Degree or higher									23%	2%	8%	12%
A‐level or equivalent									21%	8%	16%	20%
O level or equivalent									34%	33%	42%	38%
Vocational									9%	22%	14%	12%
No qualification									13%	35%	20%	18%
Smoked during pregnancy	31%	44%	42%	37%	38%	60%	51%	48%	15%	48%	33%	28%
Family characteristics at 11												
Economic circumstances												
Maternal employment	62%	72%	69%	73%	55%	46%	51%	49%	74%	47%	60%	69%
Home‐owners	47%	21%	31%	33%	67%	27%	37%	46%	81%	13%	34%	48%
Financial hardship	9%	30%	29%	38%								
Log (equivalized income)					4.16	3.95	3.92	3.81	6.34	5.75	6.04	6.17
Parental resources												
Maternal depression (severe malaise)					3%	10%	7%	5%	4%	12%	8%	8%
Aspiration: staying on at school/university	77%	68%	69%	73%	56%	47%	47%	52%	38%	31%	25%	27%
Goes to library	49%	39%	40%	38%	36%	33%	31%	31%	26%	33%	26%	21%
Plays an instrument					48%	41%	43%	42%	42%	29%	33%	33%
Goes out on walks/trips with parents	59%	44%	48%	51%	94%	86%	93%	92%				
Goes to museums	59%	57%	64%	63%	58%	50%	51%	53%				
Plays sports					57%	52%	57%	53%	58%	41%	43%	45%
Regular bedtime									91%	84%	90%	90%
Rules on time on computer									91%	88%	90%	89%
Social stress/disruptions												
Total number of schools attended	1.6	2.0	1.9	1.9					1.3	1.5	1.5	1.4
Repartnered		32%	35%	18%		38%	45%	31%		24%	31%	15%
Number of observations (unweighted in MCS)	9,831	165	411	268	7,670	315	457	491	6,549	1,147	1,727	566
% of Children (weighted in MCS)	92%	1.6%	3.9%	2.5%	85.9%	3.5%	5.1%	5.5%	60.6%	14.1%	19.7%	5.6%

Note

For the 1958 and 2000 cohorts, early childhood is defined as parental separation before age 7, in the 1970 cohort, it is separation before 5. Middle is defined as separation between age 7 and 11 in the 1958 and 2000 cohorts and between 5 and 10 in the 1970 cohort. Weighted statistics are presented for the 2000 cohort. Variable definitions are in Table S1 and mean vales for all other control variables in Table S2.

The second panel of Table 1 reports economic and parenting characteristics at age 11. Gaps in employment and income widened by family type while differences in housing tenure, a strong indicator of a families’ economic position in GB, also diverged. Single mothers were also more likely to be depressed and have fewer aspirations for their children to stay on at school (1958 and 1970 cohorts) or go to college or university (2000 cohort). Other parental inputs are also typically lower for those in single‐mother families, although changes over time are sometimes harder to interpret: for example, in the 1958 cohort single mothers were less likely to visit the library but by 2000 visits were more common. Finally, children in single‐mother families were more likely to move school.

### Results From Structural Equation Models

The first set of results from the SEMs are reported in Table 2. The first column reports the results from the regression of verbal cognitive test scores on single motherhood and the moderating and mediating variables. The coefficients on single motherhood suggest it has no significant “direct” effect on children’s cognitive outcomes; differences between the outcomes of children in single‐mother families and those of children living with both parents at age 11 can be fully explained by differences in observable characteristics in all cohorts and regardless of the child’s age at the time of parental separation. As expected, the results also show that income, home ownership, and parents’ aspirations are associated with higher cognitive attainment while financial hardship and maternal depression are associated with worse outcomes. Maternal employment shows no association with attainment. Finally, school moves are associated with lower attainment gaps for children in the 1958 cohort but by 2000 had become insignificant.

**Table 2:** Structural Equation Models of the Association Between Age 11 Cognitive Verbal Outcomes and Living With a Single Mother (1958, 1970 and 2000 Birth Cohorts)

	Verbal cognitive score	Mediating variables (measured at age 11)					
		Economic inputs			Parenting inputs		Disruption: number of school moves
		Mothers works	Financial hardship/log income^a^	Home owner	Aspirations^b^	Maternal depression	
	(1)	(2)	(3)	(4)	(5)	(6)	(7)
1958 cohort (*n* = 10,675)							
Single mother							
At birth	−.014 (.074)	.036 (.039)	.199 (.038)***	−.214 (.034)***	−.066 (.039)*		.320 (.103)***
In early childhood	−.077 (.046)*	.056 (.025)**	.191 (.024)***	−.119 (.023)***	−.053 (.023)**		.271 (.055)***
In mid childhood	−.071 (.053)	.094 (.027)***	.283 (.030)***	−.099 (.029)***	−.025 (.027)		.237 (.063)***
Mediating variables							
Mother works	.017 (.019)						
Financial hardship	−.264 (.030)***						
Home owner	.242 (.020)***						
Aspiration	.545 (.021)***						
Number of schools attended	−.050 (.010)***						
Var(e)	.799 (.009)***	.225 (.001)***	.086 (.002)***	.205 (.002)***	.172 (.002)***		.727 (.019)***
1970 cohort (*n* = 8,933)							
Single mother							
At birth	.073 (.060)	−.072 (.032)**	−.173 (.034)***	−.280 (.028)***	−.046 (.030)	.066 (.020)***	
In early childhood	−.047 (.045)	−.032 (.025)	−.190 (.025)***	−.238 (.022)***	−.038 (.024)	.032 (.013)**	
In mid childhood	.035 (.042)	−.055 (.024)**	−.332 (.024)***	−.166 (.021)***	−.022 (.022)	.023 (.011)**	
Mediating variables							
Mother works	−.006 (.020)						
Log income^a^	.190 (.027)***						
Home owner	.208 (.024)***						
Aspiration	.417 (.021)***						
Maternal depression	−.172 (.062)***						
Var(e)	.798 (.014)***	.246 (.001)***	.144 (.002)***	.181 (.002)***	.212 (.002)***	.031 (.002)***	
2000 cohort (*n* = 9,989)							
Single mother							
At birth	−.013 (.044)	−.076 (.021)***	−.248 (.013)***	−.426 (.017)***	−.014 (.018)	.045 (.013)***	.123 (.037)***
In early childhood	−.036 (.032)	−.047 (.017)***	−.117 (.010)***	−.330 (.015)***	−.048 (.015)***	.028 (.008)***	.128 (.028)***
In mid childhood	.004 (.045)	−.009 (.021)	−.072 (.014)***	−.253 (.020)***	−.035 (.021)*	.036 (.014)***	.033 (.036)
Mediating variables							
Mother works	.037 (.031)						
Log income^a^	.289 (.066)***						
Home owner	.040 (.037)						
Aspiration	.335 (.025)***						
Maternal depression	−.295 (.070)***						
Number of schools attended	−.022 (.021)						
Var(e)	.846 (.021)***	.180 (.003)***	.061 (.001)***	.135 (.003)***	.190 (.003)***	.052 (.003)***	.405 (.020)***

Note

See Table 1 for definitions of birth, early and middle single motherhood. All regressions also include controls for mothers’ characteristics (age at birth, education, not white, smoked during pregnancy and socio‐economic status); child characteristics at birth (sex, low birthweight, twin), for having older siblings and region (8 dummy variables). Sample sizes are 10,675; 8,933 and 9,989 for the 1958, 1970 and 2000 cohorts respectively.

a Controls for financial hardship (dummy variable) for the 1958 cohort, and log equivalised disposable income for the 1970 and 2000 cohorts.

b Aspirations are a binary variable for staying on beyond the school leaving age in the 1958 and 1970 cohort, and for going to university in the 2000 cohort. Robust standard errors are in parentheses.

* *p *< .10.

** *p *< .05.

*** *p *< .01.

Columns 2–7 show the association between single motherhood and the mediating variables. As expected, single‐mother families faced considerably worse economic circumstances (Columns 2–4): they had a greater risk of financial hardship (1958 cohort), lower equivalized income (1970 and 2000 cohorts), and substantially lower rates of home ownership in all cohorts. The 1958 cohort of single mothers were, after accounting for characteristics, more likely to work than other mothers when their child was 11. Among more recent cohorts the reverse is true with mothers who were single at the time of their child’s birth, or separated when their child was under 7, are less likely to be employed than otherwise similar partnered mothers. Overall, the association between the age of children at the time of parental separation and economic resources has become increasingly graded over time, with those born to single mothers seeing economic penalties increase while children whose parents separated when they were of school age saw them decline. Columns 5–7 show how single motherhood influenced parental inputs and disruptions associated with social stress. Single motherhood was consistently associated with maternal depression and more school moves but the association with aspirations was weak and typically insignificant.

Table 3 panel (i) reports the “direct” and “indirect” effects of single motherhood on cognitive outcomes and the “total” effect (also summarized in Figure 2). Direct effects, reported in panel (i), are the same as those in the first column of Table 2 and are typically small and insignificant. The indirect effect of single motherhood on children’s verbal cognitive outcomes was, on the other hand, consistently significant and often large. Among those born in 1958, indirect effects are associated with lower test scores of between −.12‐*SD* and −.16‐*SD*. For those born in 1970, indirect effect sizes are of a similar magnitude at −.11‐*SD* to −.12‐*SD*. For the 2000 cohort, however, the indirect effect of single motherhood on children’s cognitive attainment had become graded by the age at which children’s parents separated and associated with reduced attainment of −.11‐*SD*, −.08‐*SD* and −.05‐*SD* for those born to a single mother, whose parents separated before age 7, and whose parents separated between 7 and 11 respectively. Panel (ii) reports differences in effect sizes according to children’s age at the time of parental separation and tests for statistically significant differences between them. There was no evidence of statistically significant differences in the effect of single motherhood on cognitive attainment by children’s age at the time of parental separation for those born in 1958 or 1970. In the 2000 cohort, however, indirect effect sizes differed significantly by child age at time of parental separation. Panel (iii) tests for changes in effect sizes across cohorts. For children born to a single mother indirect effect sizes have not changed over the last 40 years. For children whose parents separated during early or middle childhood there is, however, a statistically significant reduction in the indirect effect of single motherhood on child outcomes between the 1958 and 2000, and 1970 and 2000, cohorts (but not between those born in 1958 and 1970). Across cohorts, reductions in economic inputs mattered most to children’s cognitive outcomes, with differences in parental inputs and school disruptions contributing only a small share of the observed deficit in attainment. Among children in the 2000 cohort, however, the indirect effect of reduced economic resources on cognitive outcomes was smaller for children whose parents separated later in their childhood.

**Table 3:** The Effect of Single Motherhood on Children’s Verbal Cognitive Attainment at Age 11 in the 1958, 1970 and 2000 Cohorts

	1958			1970			2000		
	Birth	Early	Mid	Birth	Early	Mid	Birth	Early	Mid
	(1)	(2)	(3)	(4)	(5)	(6)	(7)	(8)	(9)
(i) Estimated indirect, direct and total effects									
Indirect effect due to…									
Mother works	.001 (.001)	.001 (.001)	.002 (.002)	.000 (.002)	.000 (.001)	.000 (.001)	−.003 (.002)	−.002 (.002)	−.000 (.001)
Home owner	−.052 (.009)***	−.029 (.006)***	−.024 (.007)***	−.058 (.009)***	−.050 (.008)***	−.034 (.006)***	−.017 (.015)	−.013 (.012)	−.010 (.009)
Income^1^	−.053 (.013)***	−.050 (.009)***	−.075 (.012)***	−.033 (.008)***	−.036 (.007)***	−.063 (.011)***	−.071 (.015)***	−.034 (.008)***	−.021 (.005)***
Maternal depression				−.011 (.005)**	−.005 (.003)*	−.004 (.002)*	−.013 (.005)**	−.008 (.003)**	−.011 (.005)**
Aspirations	−.036 (.021)*	−.029 (.013)**	−.014 (.015)	−.019 (.013)	−.016 (.010)*	−.009 (.009)	−.005 (.006)	−.016 (.007)***	−.012 (.007)*
Number of schools attended	−.016 (.006)***	−.014 (.003)***	−.012 (.004)***				−.003 (.003)	−.003 (.003)	−.001 (.001)
Indirect effect	−.156 (.029)***	−.121 (.018)***	−.123 (.023)***	−.121 (.020)***	−.107 (.015)***	−.111 (.016)***	−.112 (.018)***	−.076 (.013)***	−.054 (.013)***
Direct effect	−.014 (.074)	−.077 (.046)*	−.071 (.053)	.073 (.060)	−.047 (.045)	.035 (.042)	−.013 (.044)	−.036 (.032)	.004 (.046)
Total effect (indirect + direct effect)	−.170** (.078)	−.198 (.044)***	−.193 (.053)***	−.048 (.062)	−.154 (.046)***	−.075 (.041)*	−.125 (.046)***	−.112 (.029)***	−.050 (.046)

	1958			1970			2000		
	Birth versus early	Early versus middle	Birth versus middle	Birth versus early	Early versus middle	Birth versus middle	Birth versus early	Early versus middle	Birth versus middle
(ii) Differences in effect sizes by age of separation (within cohorts)									
Indirect	−.035 (.031)	.002 (.026)	−.033 (.035)	−.015 (.023)	.004 (.018)	−.011 (.024)	.036 (.012)***	−.022 (.013)**	−.058 (.015)***
Direct	.063 (.086)	−.006 (.069)	.057 (.088)	.120 (.073)*	−.082 (.061)	.038 (.074)	.023 (.050)	−.040 (.054)	−.017 (.063)
Total	.029 (.090)	−.005 (.071)	.024 (.092)	.106 (.075)	−.078 (.063)	.027 (.075)	−.013 (.052)	−.062 (.052)	−.075 (.061)

	1958–1970			1970 versus 2000			1958 versus 2000		
	Birth	Early	Middle	Birth	Early	Middle	Birth	Early	Middle
(iii) Change in effect sizes across cohort (by age of parental separation)									
Indirect	.035 (.033)	.015 (.023)	.011 (.028)	.009 (.025)	.030 (.019)	.054 (.022)**	.044 (.032)	.045 (.022)**	.068 (.027)**
Direct	.088 (.098)	.030 (.063)	.107 (.065)*	−.086 (.076)	.012 (.055)	−.03 (.063)	.001 (.092)	.041 (.051)	.075 (.072)
Total	.122 (.099)	.045 (.065)	.118 (.067)*	−.077 (.077)	.041 (.055)	.025 (.065)	.044 (.092)	.086 (.053)	.143 (.071)**

Note

Notes as Table 2. Bootstrapped standard errors (200 repetitions) are in parentheses. Differences in coefficients and standard errors across cohorts in (iii) are obtained by estimating pairwise nested models. 1

* *p *< .10.

** *p *< .05.

*** *p *< .01.

We reproduce these results using the more detailed, but less comparable, parenting measures described above. Results are presented in Tables 4 and 5. In the 1958 cohort single motherhood was associated with a reduced probability of visiting the library and of going on outings with parents, both of which were associated with higher attainment. These differences remained for children in the 1970 cohort but were smaller. However, other parenting variables, including trips to museums and whether children played sport or a musical instrument showed only a weak association with family structure. Among children in the 2000 cohort, the relationship between single motherhood and parental inputs was weaker again: birth single mothers were, for example, more likely to visit libraries and there was little relationship between family structure and the likelihood of playing an instrument, having regular bedtimes or rules set around computer use, although children in single‐mother families were less likely to play sport. Thus, while there were some differences in parenting practices for children in the earlier cohorts, for the 2000 cohort parenting activities—with the exception of sport—were largely unaffected by single motherhood. Table 5 also shows the strength of the relationship between cognitive attainment and parenting inputs. Going to the library was strongly correlated with child outcomes in the 1958 cohort but for those born in 2000 this association has vanished. Playing an instrument and going on outings with parents is consistently associated with better outcomes. Table 5 reports the direct and indirect effects of single motherhood on verbal outcomes when the additional parenting variables are added to the models. Parenting differences contributed to attainment gaps in all cohorts but the size of the effect declined over time. In 1958, parenting deficits contributed to lower attainment of between −.05‐*SD* and −.07‐*SD*. For the 2000 cohort, parenting effects were smaller (around −.03‐*SD*). Across cohorts, economic differences were more to explaining children’s lower attainment, reducing test scores by between −.07‐*SD* and −.10‐*SD* in 1958, and −.03‐*SD* to −.09‐*SD* in 2000.

**Table 4:** Structural‐Equation Models of Cognitive Outcomes at 11, Results for Additional Measures of Parental Inputs (1958, 1970 and 2000 Birth Cohorts)

	Verbal cognitive score	Mediating variables (measured at age 11)						
		Goes to library	Plays an instrument	Out with parents	Goes to museums	Plays sports	Regular bedtime	Rules on time on computer
	(1)	(2)	(3)	(4)	(5)	(6)	(7)	(8)
1958 cohort (*n* = 10,925)								
Single mother								
At birth	.013 (.073)	−.102 (.040)**		−.154 (.042)***		−.031 (.046)		
In early childhood	−.056 (.045)	−.080 (.026)***		−.093 (.026)***		.038 (.028)		
In mid childhood	−.045 (.051)	−.097 (.030)***		−.066 (.030)**		.041 (.033)		
Mediating variables								
Goes to library	.345 (.019)***							
Out with parents (walks)	.033 (.018)*							
Plays sports (pool)	−.016 (.020)							
1970 cohort (*n* = 9,169)								
Single mother								
At birth	.085 (.058)	−.037 (.029)	−.058 (.029)**	−.062 (.021)***	−.005 (.030)	−.037 (.031)		
In early childhood	−.039 (.045)	−.042 (.023)*	−.028 (.023)	.004 (.013)	−.029 (.025)	−.011 (.025)		
In mid childhood	.048 (.042)	−.043 (.022)**	−.045 (.021)**	−.016 (.013)	−.035 (.023)	−.039 (.024)*		
Mediating variables								
Goes to library	.132 (.021)***							
Plays an instrument	.178 (.021)***							
Out with parents	.060 (.040)***							
Goes to museums	.092 (.021)							
Plays sports	−.066 (.020)***							
2000 cohort (*n* = 10,249)								
Single mother								
At birth	−.010 (.043)	.047 (.019)**	−.014 (.019)			−.074 (.021)***	−.032 (.016)**	−.013 (.013)
In early childhood	−.030 (.032)	.009 (.015)	−.006 (.016)			−.112 (.017)***	.003 (.011)	−.002 (.012)
In mid childhood	.015 (.048)	−.034 (.021)	−.044 (.027)			−.103 (.025)***	.002 (.015)	−.012 (.016)
Mediating variables								
Goes to library	.012 (.026)							
Plays an instrument	.098 (.024)***							
Plays sports	.063 (.024)***							
Regular bedtime	.006 (.046)							
Rules on time on computer	.055 (.039)							

Note

Notes as Table 2

Controls for financial hardship (dummy variable) for the 1958 cohort, and log equivalised disposable income for the 1970 and 2000 cohorts.

* *p *< .10.

** *p *< .05.

*** *p *< .01.

**Table 5:** Estimated Indirect and Direct Effects of Single Motherhood on Children’s Verbal Cognitive Attainment at Age 11 in 1958, 1970 and 2000 With Extra Measures of Parental Inputs

	1958			1970			2000		
	Birth	Early	Mid	Birth	Early	Mid	Birth	Early	Mid
	(1)	(2)	(3)	(4)	(5)	(6)	(7)	(8)	(9)
Indirect effect due to…									
Mother works	.001 (.001)	.001 (.001)	.002 (.002)	0.000 (.001)	.000 (.001)	.000 (.001)	−.003 (.002)	−.002 (.002)	−.000 (.001)
Home owner	−.048 (.010)***	−.027 (.007)***	−.022 (.006)***	−.055 (.009)***	−.047 (.007)***	−.033 (.006)***	−.018 (.015)	−.014 (.012)	−.011 (.009)
Income^a^	−.048 (.012)***	−.046 (.008)***	−.069 (.011)***	−.033 (.008)***	−.036 (.007)***	−.063 (.011)***	−.068 (.015)***	−.032 (.007)***	−.019 (.005)***
Total economic	−.097 (.015)***	−.072 (.011)***	−.089 (.014)***	−.088 (.012)***	−.083 (.010)***	−.096 (.012)***	−.089 (.015)***	−.048 (.011)***	−.030 (.009)***
Depression				−.011 (.005)**	−.005 (.003)*	−.004 (.002)*	−.013 (.006)**	−.008 (.004)**	−.010 (.005)**
Aspirations	−.033 (.020)*	−.026 (.012)**	−.012 (.012)	−.017 (.011)	−.014 (.009)	−.008 (.008)	−.005 (.006)	−.016 (.005)***	−.011 (.007)
Goes to library	−.035 (.013)***	−.028 (.009)***	−.033 (.010)***	−.005 (.004)	−.006 (.003)*	−.006 (.003)*	.001 (.001)	.000 (.000)	−.000 (.001)
Plays an Instrument				−.010 (.006)*	−.005 (.004)	−.008 (.004)**	−.001 (.002)	−.001 (.002)	−.004 (.003)*
Out with parents	−.005 (.003)	−.003 (.002)	−.002 (.002)	−.004 (.003)	.000 (.001)	−.001 (.001)			
Goes to museums				−.000 (.003)	−.003 (.002)	−.003 (.002)			
Plays sports	.000 (.001)	−.001 (.001)	−.001 (.001)	.002 (.002)	.001 (.002)	.003 (.002)	−.005** (.002)	−.007 (.003)***	−.007** (.003)
Regular bedtime							−.000 (.001)	.000 (.000)	.000 (.001)
Rules on time on computer							−.001 (.001)	−.000 (.001)	−.001 (.001)
Total parenting	−.072 (.025)***	−.058 (.016)***	−.049 (.016)***	−.045 (.016)***	−.032** (.012)	−.028 (.010)***	−.024** (.009)	−.031 (.006)***	−.033 (.011)***
Number of schools attended	−.014** (.007)	−.012 (.004)***	−.011 (.004)***				−.003 (.003)	−.003 (.003)	−.001 (.001)
Indirect effect	−.183 (.030)***	−.142 (.021)***	−.148 (.025)***	−.133 (.022)***	−.115 (.015)***	−.123 (.017)***	−.116 (.016)***	−.082 (.012)***	−.065 (.014)***
Direct effect	.013 (.071)	−.056 (.042)	−.045 (.045)	.085 (.052)	−.039 (.046)	.048 (.042)	−.010 (.045)	−.030 (.033)	.015 (.047)
Total effect (total indirect effect + direct effect)	−.170 (0.075)**	−.198 (0.046)***	−.193 (0.051)***	−.048 (0.055)	−.154 (0.046)***	−.076 (0.044)*	−.125 (0.044)***	−.113 (0.033)***	−.050 (0.046)

Note

Notes as Table 3. Bootstrapped standard errors (200 repetitions) are in parentheses.

a Controls for financial hardship (dummy variable) for the 1958 cohort, and log equivalised disposable income for the 1970 and 2000 cohorts.

* *p *< .10.

** *p *< .05.

*** *p *< .01.

The final part of our analysis looks at how repartnering influenced attainment gaps. Results are reported in Table 6. The coefficients show how repartnering influenced children’s cognitive outcome vis‐à‐vis living with a single mother. In both the 1958 and 1970 cohorts repartnering was associated with higher cognitive attainment linked to improvements in single‐mothers’ economic circumstances, but the overall effect on attainment was negative. For the 1958 cohort this was because repartnering was associated with reduced aspirations for children’s education, while for the 1970 cohort it resulted from more school moves. Among children in the 2000 cohort, neither changes in economic nor parental inputs following repartnering affected cognitive outcomes. The fact that changes in economic circumstances no longer affected cognitive outcomes may reflect changes to the tax and benefit system which, by raising the incomes of single‐parent families reduced the economic gains to repartnering. Finally, in the United States, multiple family transitions are of particular concern. We are not able to identify multiple transitions in the 1958 and 1970 birth cohorts. However, in the 2000 cohort just 2.7% (or 95) children who had lived with a single mother also experienced multiple family transitions, suggesting this is a less important phenomenon for children in GB.

**Table 6:** Estimated Direct and Indirect Effects of Re‐Partnering on Children’s Verbal Cognitive Attainment at Age 11 in 1958, 1970 and 2000

	1958	1970	2000
Indirect effect due to…			
Mother works	−.002 (.002)	−.000 (.001)	−.001 (.001)
Home owner	.016 (.009)*	.015 (.005)***	.004 (.004)
Financial hardship/income*, *, a	.032 (.010)***	.050 (.010)***	−.009 (.005)*
Maternal depression		.000 (.003)	.007 (.004)
Aspirations	−.057 (.021)***	−.018 (.012)	−.001 (.007)
Number of schools attended	−.018 (.006)***		−.004 (.004)
Total indirect effect	−.029 (.027)	.048 (.018)***	−.003 (.011)
Direct effect	−.086 (.076)	−.172 (.057)***	−.005 (.045)
Combined effect (indirect + direct)	−.115 (.083)	−.125 (.055)**	−.009 (.048)

Note

Notes as Table 3. Coefficients show how cognitive outcomes change if single mothers re‐partner. Bootstrapped standard errors (200 repetitions) are in parentheses.

a Controls for financial hardship (dummy variable) for the 1958 cohort, and log equivalised disposable income for the 1970 and 2000 cohorts.

* *p *< .10.

** *p *< .05.

*** *p *< .01.

### Robustness Checks: Selection Bias, Outcome Measures, and Time Since Separation

Our results may be sensitive to our choice of outcome measure, route of entry into single motherhood, definition of timing of single motherhood or sample selectivity bias. To address these concerns, we perform a range of robustness tests. First, to test whether our results are sensitive to our chosen instrument (verbal cognitive ability) we reproduced the same analysis on normalized math and reading test scores. To ensure comparability across cohorts only results for England are reported (Table S3). Results for reading and mathematics are similar to those for verbal test scores, although consistent with other studies where there are differences, they are typically larger for mathematics (Sanz‐de‐Galdeano & Vuri, 2007).

Two further robustness checks explored how prior circumstances influenced outcomes. First, we investigated how the route of entry into single motherhood influenced outcomes. Table S4 reports results for children whose mothers were widowed or, for the 2000 cohort, had separated from a cohabiting or married partner (among earlier cohorts, cohabitation was rare). Children in the 1958 and 1970 cohorts whose mothers were widowed performed better than those in otherwise similar “intact” families. This result was partly driven by higher rates of home ownership among widowed single‐mother families. This was not seen among children with widowed mothers in the 2000 cohort, for whom there was no significant difference in attainment (vis‐à‐vis children in couples). For parents who separated, attainment deficits were significant but there was little difference between those whose parents had previously been married or cohabited. Second, we tested whether children whose parents had separated more recently had worse outcomes (Table S5) but found no evidence that test scores were more adversely affected. Another concern is that for the 1970 cohort early and middle single motherhood is defined for parental separation between 0/5 and 5/10 (rather than 0/7 and 7/11 in the other cohorts). In order to test the sensitivity of our results to this, for the 2000 cohort we examined data for ages 0/5 and 5/11. Results, in Table S6, are similar to those in Table 3.

Finally, as sample selection bias remains a concern, we replicated Table 3 including lagged dependent variables for cognitive attainment and previous economic and parental inputs as described in the methods section. Results with controls for prior attainment and characteristics at age 7 are reported in Table S7. The “direct” effect of single motherhood on children’s verbal cognitive outcomes is insignificant in all cohorts, both with and without controls for prior attainment. “Indirect” effects, on the other hand, are negative and significant. Adding controls for prior attainment leads to a small reduction in the indirect effect sizes, suggesting attainment and resources were lower prior to parental separation. For children in the 2000 cohort cognitive attainment at age 3 is also available which allowed us to consider how selectivity bias affects estimates for children whose parents separate between 3 and 7 (Tables S6, cols 7–8). As before, including controls for prior attainment reduced the size of the estimated effects, although they remained significant. Thus, while sample selection bias accounted for some of the difference in test scores between children in single and two‐parent families it cannot fully explain the difference. This is in line with Cheng et al.’s (2006) study of the effect of parental separation on children’s behavioral and emotional outcomes at 81 months which concluded that preseparation differences accounted for a small or trivial part of observed differences.

## Discussion

Prior studies suggest that single motherhood bears a weak relationship with children’s cognitive attainment once family characteristics are accounted for. The aim of this study was to get a fuller picture of this relationship using SEM to account for the mediating effect single motherhood has on the resources available to children, and the likelihood that children face other disruptions such as home or school moves. We examined changes in this relationship over 40 years and investigated differences in outcomes according to children’s age at the time of parental separation. For children born to single mothers, our results suggest that the relationship between single motherhood and children’s outcomes has remained remarkably stable over time. We also show that those children born to single mothers have become increasingly negatively selected on observable characteristics and that this has led to a growing gap in the attainment of children in single‐mother families and those living with both parents. Once we account for differences in parent and child characteristics, observed at the time of birth, the estimated effect of single motherhood on attainment, although significant, is considerably reduced and exhibits little change over time. That the relationship between children's cognitive outcomes and single motherhood has remained stable over time runs contrary to the expectation that increased prevalence, by reducing the stigma associated with single motherhood, may be associated with fewer negative consequences. For those experiencing later parental separation, however, the damaging impact of single motherhood has lessened; for those whose parents separate when they are of school age attainment gaps, both before and after adjusting for characteristics at birth, have shown significant falls.

We then partitioned the “total” effect of single motherhood on child outcomes—measured as the deficit in attainment found after adjusting for characteristics observed at birth—into a “direct effect” and “indirect” effect. Consistent with previous studies which find that single motherhood is only weakly associated with children’s cognitive outcomes once contemporary characteristics, such as income and maternal mental health, are accounted for (Chapple, 2009; Ely et al., 1999), we find that the direct effect of single motherhood on children’s cognitive attainment is small and insignificant for all cohorts, and for children of all ages at the time of parental separation. The “indirect” effects of single motherhood on children’s outcomes, mediated by reduced economic and parental inputs and social stress are, on the other hand, consistently negative and statistically significant. For those born in 1958 and 1970, the indirect effect of single motherhood on children’s verbal cognitive test scores ranged from −.11‐*SD* to −.16‐*SD* with effects showing little variation by the time of parental separation. In the 2000 cohort, indirect effect sizes remained large (−.11‐*SD*) for children born to single mothers but fell among those whose parents separated during early or middle childhood (−.07‐*SD* and −.05‐*SD* respectively). As a result, by 2000 indirect effects had become increasingly age graded, with children who were younger at the time of parental separation having larger deficits.

To understand the indirect effect of single motherhood on child outcomes we examined its impact on economic and parenting “inputs.” For parental inputs, we find that while there was an association between single motherhood and parental inputs at age 11 for children in the 1958 cohort, this association has declined sharply over time. For children born in 2000 there is little significant relationship between single motherhood and parenting practices. This is in line with US evidence which, using longitudinal data and fixed effect models, showed that changes in family structure had little impact on maternal parenting behavior (Gibson‐Davis, 2008). On the other hand, across cohorts, single motherhood is consistently associated with reduced economic resources. Moreover, in the 2000 cohort single motherhood had a considerably greater impact on the incomes of children born to single mothers than those experiencing later parental separation. As a consequence, the “indirect” effect of reduced income on attainment was greater for children born to single mothers, a result not found in the earlier cohorts. Putting together our results on how single motherhood affects economic and parental inputs, and the importance of these inputs to cognitive outcomes, we show that single‐mother families’ weaker economic position is the most important factor for explaining the lower cognitive test‐scores of their children. The role of parental inputs in mediating lower attainment is small and has become less important over time. Overall our results show striking similarities to those of Thomson, Hanson, and McLanahan’s (1994) for the United States, who reported that losses in income accounted for up to half of the negative consequences of single motherhood for child outcomes while parenting behavior played a minor role in explaining disadvantage.

The final stage of our analysis investigated whether repartnering led to changes in children’s cognitive outcomes. In the early cohorts repartnering was associated with lower cognitive test scores, vis‐à‐vis single motherhood, because, while families’ economic circumstances improved, parental inputs declined and the (negative) direct effect of repartnering grew. The association between repartnering and children’s cognitive outcomes has changed over time; in the 2000 cohort it had no effect on child outcomes either through direct or mediating channels. This echoes recent UK evidence from the 2000 cohort showing children in stepfather families have similar cognitive outcomes to those living continuously with a single mother (Mariani, Ozcan, & Goisis, 2017). An important question for future research, however, is whether behavior has changed as the pressure to repartner has declined (e.g., because of reforms to the welfare system, or a reduction in stigma associated with single motherhood). In addition, we have not examined multiple family transitions, a topic which has attracted growing attention in the United States. The United States differs to European countries, however, in its patterns of family formation; Fomby, Cavanagh, and Goode’s (2011) comparative study of the United States and United Kingdom found repartnering and social fathering to be less common in the United Kingdom, a result supported by our data on multiple family transitions for children born in 2000.

While our analysis provides important new evidence on the cognitive attainment of children in single‐mother families, several limitations remain. First, we look only at how single motherhood influences children’s cognitive attainment. Studies typically show larger effects for children’s emotional outcomes (Cheng et al., 2006; Mariani et al., 2017) and parental inputs may have a stronger impact on these outcomes (Carlson & Corcoran, 2001). Moreover, in contrast to our findings, Fitzsimons and Villadsen (2019), using data from the 2000 cohort, show that later parental separations are more damaging to children’s mental health than those in early childhood. Second, our measured “inputs” are incomplete and may suffer from important omitted variable bias. Specifically, we have not been able to control for child effects, although evidence suggests that children’s characteristics may influence the likelihood of parental separation; for example, parents of children with attention deficit hyperactivity disorder are more likely to separate (Kvist, Nielsen, & Simonsen, 2013). If these omitted variables are correlated with family status our estimates will be biased. Third, while we examined selection bias among those whose parents separated after age 3, we are not able to account for potential selection bias among children who were born to single mothers or whose parents separated when they were very young. For this group selection bias may remain a problem. Fourth, comparability across surveys is limited by data availability and the quality of parenting measures. Finally, while we touched on the effect repartnering has on children’s cognitive outcomes, we have not considered how children’s relationships with an absent father, such as father contact or payment of maintenance, influences their outcomes. All of these are important issues for future research.

This article has provided new evidence on the association between single motherhood and children’s cognitive attainment. We show that while single motherhood has become much more common it still has, on average, negative consequences for children’s cognitive attainment because it reduces the resources available to them. Today almost all of the relationship between single motherhood and children’s verbal cognitive outcome is explained by families’ reduced economic circumstances. Deficits associated with parenting having all but disappeared over the last 40 years. Over time, we have also seen the effect of parental separation on child outcomes become increasingly graded by the age at which parents separate. Between 1958 and 2000 there was no significant change in the cognitive attainment gaps of children born to single mothers vis‐à‐vis those living with both parents. However, for those whose parents separated when they were of school age these deficits declined largely because mothers who separated when their children were older suffered a smaller reduction in their economic well‐being. Overall the findings suggest two policy responses: supporting the incomes of single‐parent families, and in particular those with very young children, and addressing the growing gap in attainment between all children whose parents have adverse economic characteristics, whether partnered or not.

## Supporting information


**Table S1.** Variable Definitions

**Table S2.** Descriptive Statistics (Additional Control Variables)

**Table S3.** England Only Estimates for Mathematics and Reading Test Scores

**Table S4.** Estimated Indirect and Direct Effects of Widowhood and Separation From Cohabitation or Marriage on Children’s Verbal Cognitive Attainment at Age 11 in 1958, 1970 and 2000

**Table S5.** Direct and Indirect Effect of Parental Separation in “Middle” Childhood by Years Since Separation on Verbal Cognitive Ability (1970 and 2000 Cohort)

**Table S6.** Robustness Checks of the Estimated Indirect and Direct Effects of Single Motherhood During Early (Age 0–5) and Middle (5–11) Childhood on Verbal Cognitive Attainment, 2000 Cohort

**Table S7.** Association of Single Motherhood With Verbal Cognitive Outcomes at Age 11 for Those Whose Parents Separate in Middle Childhood, With and Without Controls for Prior Attainment (at 5 or 7)

## Footnotes

1 Controls for financial hardship (dummy variable) for the 1958 cohort, and log equivalised disposable income for the 1970 and 2000 cohort.
