# Supplementary material for: The Rise in Single‐Mother Families and Children’s Cognitive Development: Evidence From Three British Birth Cohorts
Source: Child Dev. 2019 Nov 20;91(5):1762–85. doi: 10.1111/cdev.13342 (PMC9328442; doi:10.1111/cdev.13342)
Supplement: Supplementary file 1 — Table S1. Variable Definitions [file CDEV-91-1762-s002.docx]

Table A1: Variable definitions

|  | 1958 | 1970 | 2000 |
| --- | --- | --- | --- |
| Child Outcomes | | | |
| *Cognitive measures- Verbal outcomes* | | | |
| Age 3 | - | - | British Abilities Scale  Naming vocabulary |
| Age 5/ 7 | Southgate Group Reading Test | English Picture Vocabulary Test at Age 5 | British Abilities Scale Word reading |
| Age 10/11 | Verbal Ability section of the General Ability test | British Abilities Scale Verbal Similarities | British Abilities Scale Verbal Similarities |
| *Family Structure Variables* | | | |
| Became single mother at birth, during early childhood (between birth and 5 /7), or middle childhood (between 5/7 and 10/11). | | | |
| *Child characteristics, sibling information and area at birth* | | | |
| *Child information:* Gender, low weight at birth (less than 2.5 kg), dummy variable for non-white  *Sibling information*: for the 1958 Cohort, previous births take the value of 1 if the biological mother reports a positive length of time between cohort child’s birth and previous birth and 0 otherwise; for 1970 and 2000, we control for the number of older siblings)  *Region*: region of residence at birth (11 standard regions) | | | |
| *Mother Characteristics at birth* | | | |
| *Age:* Two dummy variables for mother over 30 or under 21 at birth  *Education dummies:* for the 1958 cohort, dummy for left school at or before minimum leaving age (age 14 or 15, depending on maternal age); for the 1970 cohort dummy variables for left education at minimum leaving age (age 15) or earlier and at 19 or older; for the 2000 cohort, highest qualification obtained (no qualification or GCSE D-D, O level/GCSE A-C, A/AS/S levels or diploma, degree or higher education, other education,).  *Mothers social class at birth*: mother's social class at birth, based on current or last occupation.  *Smoking:* whether mother smoked during pregnancy. | | | |
| *Economic circumstances at 11* | | | |
| In Work | Mother is in work at 10 (11) | | |
| House-Owners | Owner occupied accommodation | | |
| Net weekly family income / financial hardship | Takes the value of 1 if the family went through financial difficulties last year. | Generated by conducting an interval regression on the banded gross family income at 10 and applying different tax rates according to the gross income reported to generate net weekly income. Income is then equivalised using the square root of household scale. | Equivalised (using the modified OECD scale) weekly net family income. |
| *Maternal Mental Health* | | | |
| Severe maternal malaise at child age 11 | Not Available | Severe malaise derived from 24 item Rutter Scale. Those with scores above the 95^th^ percentile are defined as ‘severe malaise.’^[[1]](#footnote-1)^ | Severe malaise derived from the Kessler scale, with scores >13 defined as severe. |
| *Parenting at 11* |  |  |  |
| Stay on at school / college/ university | Parent hopes child will stay at school beyond minimum age | Mother thinks child would leave school at 17 or 18 versus don't know or 16. | Mother thinks child is very likely to attends university. |
| Goes to library | Went often to the public library in the 12 months previous to the age 11 interview | Goes to the library often as a spare time activity | Visits library at least once a month. |
| Plays an instrument |  | Plays musical instruments often or sometimes as a spare time activity | Whether plays a musical instrument |
| Goes on outings with parent(s) | Most weeks goes for a visit or walk with the mum or dad | Goes out, has meals or goes for walks with family often |  |
| Goes to museums |  | Goes to museums sometimes or often as an activity during spare time. |  |
| Plays sports | Went often to the swimming pool in the 12 months previous to the age 11 interview | Conducts sports often as a spare time activity | Plays sports or does exercise at least twice a week |
| Regular bedtime |  |  | Regular bedtime: usually or always a regular bedtime |
| Rules on time on computer |  |  | Has rules on computer times |
| *Disruptions by 11* | | | |
| Number of schools attended | Number of schools attended (asked at 11) | Not available | Number of school attended between age 5 to 11 |

1. See <http://doc.ukdataservice.ac.uk/doc/5805/mrdoc/pdf/MotherMalaiseQuestions.pdf> [↑](#footnote-ref-1)
