# Supplementary material for: The Rise in Single‐Mother Families and Children’s Cognitive Development: Evidence From Three British Birth Cohorts
Source: Child Dev. 2019 Nov 20;91(5):1762–85. doi: 10.1111/cdev.13342 (PMC9328442; doi:10.1111/cdev.13342)
Supplement: Supplementary file 2 — Table S2. Descriptive Statistics (Additional Control Variables) [file CDEV-91-1762-s006.docx]

Table A2: Descriptive statistics (additional control variables)

|  | Both biological parents  at 11 | Single mothers at birth | Single mothers, early childhood | Single mothers, middle  childhood | Both biological parents  at 11 | Single mothers at Birth | Single mothers, early childhood | Single mothers, middle  childhood | Both biological parents  at 11 | Single mothers at Birth | Single mothers, early childhood | Single mothers, middle  childhood |
| --- | --- | --- | --- | --- | --- | --- | --- | --- | --- | --- | --- | --- |
|  | 1958 cohort | | | | 1970 cohort | | | | 2000 cohort | | | |
| Child and family characteristics |  |  |  |  |  |  |  |  |  |  |  |  |
|  | (1) | (2) | (3) | (4) | (5) | (6) | (7) | (8) | (9) | (10) | (11) | (12) |
| Male | 51% | 42% | 51% | 47% | 51% | 43% | 51% | 54% | 50% | 51% | 54% | 48% |
| Low birth weight | 3% | 9% | 6% | 6% | 5% | 8% | 7% | 5% | 6% | 8% | 7% | 7% |
| Has older sibling | 64% | 41% | 68% | 68% |  |  |  |  |  |  |  |  |
| Number older siblings |  |  |  |  | 1.1 | 0.8 | 1.2 | 1.1 | 0.9 | 0.8 | 0.9 | 1.1 |
| Mother Characteristics at birth |  |  |  |  |  |  |  |  |  |  |  |  |
| *Socio-economic class (based on current or last job)* |  |  |  |  |  |  |  |  |  |  |  |  |
| Higher / Managerial or professional | 5% | 4% | 4% | 3% | 9% | 5% | 9% | 11% | 37% | 7% | 18% | 24% |
| Intermediate | 21% | 37% | 21% | 25% | 37% | 40% | 36% | 38% | 20% | 11% | 15% | 20% |
| Lower / Lower supervisory + routine/semi-routine | 12% | 37% | 16% | 11% | 20% | 44% | 26% | 22% | 33% | 62% | 55% | 47% |
| Unemployed / retired / housekeeper | 63% | 22% | 59% | 62% | 34% | 11% | 29% | 29% |  |  |  |  |
| Small employer / self employed |  |  |  |  |  |  |  |  | 5% | 2% | 4% | 5% |
| *Ethnicity* |  |  |  |  |  |  |  |  |  |  |  |  |
| Not white | 1% | 8% | 1% | 2% | 2% | 8% | 2% | 3% | 15% | 23% | 12% | 7% |
