# Supplementary material for: The Rise in Single‐Mother Families and Children’s Cognitive Development: Evidence From Three British Birth Cohorts
Source: Child Dev. 2019 Nov 20;91(5):1762–85. doi: 10.1111/cdev.13342 (PMC9328442; doi:10.1111/cdev.13342)
Supplement: Supplementary file 3 — Table S3. England Only Estimates for Mathematics and Reading Test Scores [file CDEV-91-1762-s005.docx]

Appendix A3: England only estimates for Mathematics and Reading Test Scores

1. Mathematics

|  | Birth | Early | Mid | Birth | Early | Mid | Birth | Early | Mid |
| --- | --- | --- | --- | --- | --- | --- | --- | --- | --- |
|  |  | 1958 |  |  | 1970 |  |  | 2000 |  |
| Mother Works | .000 | .000 | .000 | .002 | .001 | .001 | -.004 | -.003 | -.002 |
|  | (.001) | (.001) | (.001) | (.002) | (.001) | (.001) | (.004) | (.002) | (.002) |
| Home owner | -.066*** | -.033*** | -.040*** | -.078*** | -.058*** | -.042*** | -.059*** | -.043*** | -.036*** |
|  | (.014) | (.008) | (.010) | (.011) | (.009) | (.008) | (.020) | (.015) | (.012) |
| Income^1^ | -.052*** | -.052*** | -.077*** | -.032*** | -.032*** | -.054*** | -.023 | -.010 | -.007 |
|  | (.012) | (.009) | (.012) | (.008) | (.008) | (.011) | (.017) | (.008) | (.005) |
| Maternal depression |  |  |  | -.005 | -.002 | -.001 | -.005 | -.003 | -.006* |
|  |  |  |  | (.005) | (.002) | (.001) | (.003) | (.002) | (.004) |
| Aspirations | -.035 | -.027** | -.018 | -.037** | -.010 | -.004 | .000 | -.018** | -.022* |
|  | (.022) | (.014) | (.016) | (.016) | (.013) | (.011) | (.014) | (.008) | (.012) |
| Number of schools | -.017** | -.014*** | -.010** |  |  |  | -.011** | -.007** | -.005 |
| attended | (.007) | (.004) | (.004) |  |  |  | (.005) | (.003) | (.004) |
| Total Indirect Effect | -.169*** | -.126*** | -.145*** | -.150*** | -.103*** | -.1*** | -.101*** | -.085*** | -.076*** |
|  | (.031) | (.021) | (.026) | (.024) | (.020) | (.017) | (.023) | (.015) | (.018) |
| Direct Effect | -.033 | -.065 | -.088 | -.074 | -.110** | -.017 | -.105* | -.082** | .017 |
|  | (.071) | (.048) | (.057) | (.062) | (.048) | (.043) | (.056) | (.041) | (.055) |
| Combined effect (total | -.202*** | -.191*** | -.233*** | -.224*** | -.213*** | -.117** | -.206*** | -.167*** | -.060 |
| indirect effect + direct effect) | (.070) | (.047) | (.059) | (.064) | (.048) | (.044) | (.056) | (.041) | (.056) |

1. Reading

|  | Birth | Early | Mid | Birth | Early | Mid | Birth | Early | Mid |
| --- | --- | --- | --- | --- | --- | --- | --- | --- | --- |
|  |  | 1958 |  |  | 1970 |  |  | 2000 |  |
| Mother Works | .001 | .000 | .001 | .000 | .000 | .000 | -.000 | -.000 | -.000 |
|  | (.001) | (.001) | (.002) | (.003) | (.001) | (.001) | (.003) | (.002) | (.001) |
| Home owner | -.057*** | -.028*** | -.035*** | -.075*** | -.056*** | -.041*** | .000 | .000 | .000 |
|  | (.012) | (.008) | (.009) | (.012) | (.009) | (.007) | (.018) | (.013) | (.011) |
| Income^1^ | -.047*** | -.047*** | -.070*** | -.024*** | -.025*** | -.041*** | -.063*** | -.029*** | -.019*** |
|  | (.011) | (.009) | (.012) | (.007) | (.007) | (.010) | (.017) | (.008) | (.007) |
| Maternal depression |  |  |  | -.007 | -.003 | -.002 | -.007 | -.004 | -.008* |
|  |  |  |  | (.005) | (.003) | (.001) | (.004) | (.003) | (.004) |
| Aspirations | -.036* | -.025* | -.018 | -.042** | -.013 | -.006 | -.001 | -.017** | -.021* |
|  | (.021) | (.014) | (.016) | (.019) | (.015) | (.012) | (.013) | (.008) | (.012) |
| Number of schools | -.017*** | -.013*** | -.010** |  |  |  | -.004 | -.003 | -.002 |
| attended | (.007) | (.004) | (.004) |  |  |  | (.003) | (.002) | (.002) |
| Total Indirect Effect | -.156*** | -.114*** | -.131*** | -.148*** | -.097*** | -.089*** | -.074*** | -.052*** | -.050*** |
|  | (.030) | (.021) | (.025) | (.025) | (.020) | (.018) | (.024) | (.014) | (.016) |
| Direct Effect | -.012 | -.052 | .006 | .028 | -.044 | -.011 | -.020 | -.067 | -.008 |
|  | (.078) | (.050) | (.059) | (.060) | (.044) | (.046) | (.057) | (.042) | (.060) |
| Combined effect (total | -.168* | -.166*** | -.125* | -.120* | -.141*** | -.100** | -.094* | -.119*** | -.057 |
| indirect effect + direct effect) | (.089) | (.051) | (.064) | (.064) | (.044) | (.047) | (.054) | (.042) | (.060) |

Notes: As Table 3. Sample sizes are 8811 for the 1958 cohort, 7559 for the 1970 cohort and 6,071 for maths and 6056 for reading for the 2000 cohort.
