# Supplementary material for: The Rise in Single‐Mother Families and Children’s Cognitive Development: Evidence From Three British Birth Cohorts
Source: Child Dev. 2019 Nov 20;91(5):1762–85. doi: 10.1111/cdev.13342 (PMC9328442; doi:10.1111/cdev.13342)
Supplement: Supplementary file 4 — Table S4. Estimated Indirect and Direct Effects of Widowhood and Separation From Cohabitation or Marriage on Children’s Verbal Cognitive Attainment at Age 11 in 1958, 1970 and 2000 [file CDEV-91-1762-s004.docx]

Table A4: Estimated indirect and direct effects of widowhood and separation from cohabitation or marriage on children’s verbal cognitive attainment at age 11 in 1958, 1970 and 2000

|  | 1958 | 1970 | 2000 | 2000 | 2000 |
| --- | --- | --- | --- | --- | --- |
|  | Widow | Widows | Widows | Previously  married | Previously  cohabiting |
| Mother Works | .001 | .000 | -.006 | -.003 | -.001 |
|  | (.002) | (.002) | (.005) | (.002) | (.003) |
| Home owner | .034*** | .042*** | -.004 | -.017 | -.011 |
|  | (.010) | (.011) | (.004) | (.015) | (.016) |
| Income^1^ | .048*** | -.013 | -.025** | -.039*** | -.020*** |
|  | (.011) | (.011) | (.011) | (.008) | (.005) |
| Maternal depression |  | .003 | -.014 | -.006 | -.010*** |
|  |  | (.005) | (.016) | (.004) | (.003) |
| Aspirations | .034* | .004 | -.076*** | -.016** | -.0012** |
|  | (.019) | (.020) | (.017) | (.006) | (.007) |
| Number of schools | .016*** |  | .004 | -.002 | -.003 |
| attended | (.005) |  | (.004) | (.002) | (.002) |
| Total Indirect Effect | .134*** | .037 | -.122*** | -.082*** | -.058*** |
|  | (.027) | (.031) | (.028) | (.016) | (.012) |
| Direct Effect | .018 | .225** | .072 | -.010 | -.028 |
|  | (.074) | (.097) | (.125) | (.044) | (.033) |
| Combined effect (total | .152** | .263*** | -.050 | -.092** | -.086** |
| indirect effect + direct effect) | (.076) | (.101) | (.123) | (.042) | (.036) |

Notes as table 2. Total sample sizes as Table 2 for the 1958 and 1970 cohort, and 9823 for the 2000 cohort. The number of widows were 206, 100 and 62 in the 1958, 1970 and 2000 cohorts respectively. The numbers that were previously cohabiting and previously married in the 2000 cohort were 1120 and 845 respectively.
