# Supplementary material for: The Rise in Single‐Mother Families and Children’s Cognitive Development: Evidence From Three British Birth Cohorts
Source: Child Dev. 2019 Nov 20;91(5):1762–85. doi: 10.1111/cdev.13342 (PMC9328442; doi:10.1111/cdev.13342)
Supplement: Supplementary file 5 — Table S5. Direct and Indirect Effect of Parental Separation in “Middle” Childhood by Years Since Separation on Verbal Cognitive Ability (1970 and 2000 Cohort) [file CDEV-91-1762-s001.docx]

Table A5: Direct and indirect effect of parental separation in ‘middle’ childhood by years since separation on verbal cognitive ability (1970 and 2000 cohort)

|  | Without Prior Attainment | | | | With Prior Attainment | | | |
| --- | --- | --- | --- | --- | --- | --- | --- | --- |
|  | 1 year ago | 2 years ago | 3 years ago | 4 years ago | 1 year ago | 2 years ago | 3 years ago | 4 years ago |
|  | (1) | (2) | (3) | (4) | (5) | (6) | (7) | (8) |
| 1970 cohort |  |  |  |  |  |  |  |  |
| Total Indirect Effect | -.127*** | -.123*** | -.097*** | -.114*** | -.093*** | -.086*** | -.066*** | -.070*** |
|  | (.033) | (.032) | (.034) | (.023) | (.025) | (.022) | (.026) | (.017) |
| Direct Effect | .070 | -.011 | -.043 | .076 | .044 | .038 | -.038 | .068 |
|  | (.088) | (.097) | (.108) | (.075) | (.085) | (.093) | (.099) | (.073) |
| Total Effect | -.057 | -.133 | -.141 | -.038 | -.050 | -.048 | -.104 | -.002 |
|  | (.092) | (.097) | (.109) | (.078) | (.086) | (.090) | (.101) | (.076) |
| 2000 Cohort |  |  |  |  |  |  |  |  |
| Total Indirect Effect | -.028 | -.048*** | -.018 | -.064*** | -.023* | -.034*** | -.013 | -.040*** |
|  | (.018) | (.018) | (.018) | (.018) | (.012) | (.013) | (.012) | (.013) |
| Direct Effect | -.053 | .036 | -.005 | -.013 | -.044 | .045 | .008 | -.022 |
|  | (.085) | (.101) | (.076) | (.089) | (.082) | (.097) | (.075) | (.083) |
| Total effect | -.081 | -.012 | -.023 | -.077 | -.067 | .012 | -.004 | -.063 |
|  | (.085) | (.101) | (.075) | (.089) | (.081) | (.096) | (.074) | (.082) |

Notes: As table 2. Sample sizes are 8,161 for the 1970 cohort and 7,115, for the 2000 cohort.
