# Supplementary material for: The Rise in Single‐Mother Families and Children’s Cognitive Development: Evidence From Three British Birth Cohorts
Source: Child Dev. 2019 Nov 20;91(5):1762–85. doi: 10.1111/cdev.13342 (PMC9328442; doi:10.1111/cdev.13342)
Supplement: Supplementary file 6 — Table S6. Robustness Checks of the Estimated Indirect and Direct Effects of Single Motherhood During Early (Age 0–5) and Middle (5–11) Childhood on Verbal Cognitive Attainment, 2000 Cohort [file CDEV-91-1762-s007.docx]

Table A6: Robustness checks of the estimated indirect and direct effects of single motherhood during early (age 0-5) and middle (5-11) childhood on verbal cognitive attainment, 2000 cohort

|  | Birth | Early (0-5) | Mid (5-11) |
| --- | --- | --- | --- |
| Mother Works | -0.003 | -0.002 | -0.001 |
|  | (0.002) | (0.002) | (0.001) |
| Home owner | -0.017 | -0.013 | -0.012 |
|  | (0.016) | (0.012) | (0.011) |
| Income^1^ | -0.071*** | -0.036*** | -0.023*** |
|  | (0.016) | (0.009) | (0.006) |
| Maternal depression | -0.013** | -0.010*** | -0.007** |
|  | (0.006) | (0.004) | (0.003) |
| Aspirations | -0.005 | -0.020*** | -0.009 |
|  | (0.006) | (0.006) | (0.006) |
| Number of schools | -0.003 | -0.003 | -0.002 |
| attended | (0.003) | (0.003) | (0.002) |
| Total Indirect Effect | -0.112*** | -0.065*** | -0.053*** |
|  | (0.017) | (0.035) | (0.012) |
| Direct Effect | -0.016 | -0.083*** | 0.026 |
|  | (0.044) | (0.012) | (0.037) |
| Combined effect (total | -0.128*** | -0.148*** | -0.027 |
| indirect effect + direct effect) | (0.046) | (0.035) | (0.037) |
|  |  |  |  |

Notes: as Table 2. Sample sizes as Table 2.
