# Supplementary material for: The Rise in Single‐Mother Families and Children’s Cognitive Development: Evidence From Three British Birth Cohorts
Source: Child Dev. 2019 Nov 20;91(5):1762–85. doi: 10.1111/cdev.13342 (PMC9328442; doi:10.1111/cdev.13342)
Supplement: Supplementary file 7 — Table S7. Association of Single Motherhood With Verbal Cognitive Outcomes at Age 11 for Those Whose Parents Separate in Middle Childhood, With and Without Controls for Prior Attainment (at 5 or 7) [file CDEV-91-1762-s003.docx]

Table A7: Association of single motherhood with verbal cognitive outcomes at age 11 for those whose parents separate in middle childhood, with and without controls for prior attainment (at 5 or 7)

|  | Separation in Middle Childhood | | | | | | Separation between 3 and 7 | | |
| --- | --- | --- | --- | --- | --- | --- | --- | --- | --- |
|  | 1958 |  | 1970 |  | 2000 |  | 2000 | |  |
|  | Without prior  attainment | With prior  attainment | Without prior  attainment | With prior  attainment | Without prior  attainment | With prior  attainment | Without prior  attainment | With prior  attainment | |
| Direct Effect | -0.066 | -0.029 | 0.035 | 0.038 | -0.006 | -0.002 | -0.061 | -0.059 | |
|  | (0.053) | (0.045) | (0.042) | (0.041) | (0.047) | (0.046) | (0.050) | (0.046) | |
| Indirect Effect | -0.128*** | -0.041*** | -0.110*** | -0.074*** | -0.043*** | -0.029*** | -0.072*** | -0.036** | |
|  | (0.023) | (0.010) | (0.018) | (0.012) | (0.012) | (0.009) | (0.017) | (0.014) | |
| Total Effect | -0.194*** | -0.070* | -0.075 | -0.036 | -0.049 | -0.031 | -0.133*** | -0.095** | |
|  | (0.057) | (0.042) | (0.047) | (0.038) | (0.047) | (0.046) | (0.044) | (0.041) | |

Note: Note the coefficients without controls for prior attainment are very similar, but not identical, to those in Table 4 because the sample is restricted to include whose parents separate in mid childhood and couples. The lagged dependent variable is age 7 reading attainment for the 1958 cohort and age 5 vocabulary scores for the 1970 and 2000 cohorts. For those whose parents separate in mid childhood, the sample sizes were 10,099 for the 1958 cohort, 8,161 for the 1970 cohort and 7,115 for the 2000 cohort. For the 2000 cohort, for children whose parents separate between 3 and 7 the sample size is 7,367.
